# Factors associated with adherence to noninvasive positive pressure ventilation in amyotrophic lateral sclerosis

**DOI:** 10.1371/journal.pone.0302515

**Published:** 2024-05-15

**Authors:** Hee Soo Kim, Hyeonseong Woo, Seok-Jin Choi, Jong-Gyu Baek, Ju Seok Ryu, Hyung-Ik Shin, Kyung Seok Park, Jaewon Beom

**Affiliations:** 1 Department of Rehabilitation Medicine, Seoul National University College of Medicine, Seoul National University Bundang Hospital, Seoul, Republic of Korea; 2 Department of Neurology, Seoul National University College of Medicine, Seoul National University Hospital, Seoul, Republic of Korea; 3 Department of Neurology, Yeungnam University College of Medicine, Daegu, Republic of Korea; 4 Department of Rehabilitation Medicine, Seoul National University College of Medicine, Seoul National University Hospital, Seoul, Republic of Korea; 5 Department of Neurology, Seoul National University College of Medicine, Seoul National University Bundang Hospital, Seoul, Republic of Korea; University Medical Centre Ljubljana (UMCL) / Faculty of Medicine, University Ljubljana (FM,UL), SLOVENIA

## Abstract

**Introduction:**

This cohort study aimed to investigate the factors associated with noninvasive positive pressure ventilation adherence and assess the long-term effects of noninvasive positive pressure ventilation adherence in patients with amyotrophic lateral sclerosis (ALS).

**Methods:**

The medical records of patients with ALS admitted to a tertiary hospital for noninvasive positive pressure ventilation initiation were retrospectively reviewed. Pulmonary function parameters, variables of blood gas analysis, the site of symptom onset, the time from onset and diagnosis to noninvasive positive pressure ventilation application, ALS Functional Rating Scale-Revised, neurophysiological index, and the length of hospital stay were evaluated. The adherence to noninvasive positive pressure ventilation was defined as the use of noninvasive positive pressure ventilation for ≥ 2 h/day or ≥ 4 h/day. The correlations between noninvasive positive pressure ventilation adherence or length of hospital stay and other clinical parameters were analyzed.

**Results:**

Fifty-one patients with ALS were included in the study. The time from onset and diagnosis to NIPPV application was reduced by 16 months in the adherent group than that in the non-adherent group; however, the parameters of blood gas analysis and pulmonary function tests did not differ significantly between the groups. Furthermore, the neurophysiological index of the abductor digiti minimi muscle was higher by 4.05 in the adherent group than that in the non-adherent group. The adherence to noninvasive positive pressure ventilation prolonged tracheostomy-free survival compared to that of non-adherence. Desaturation events, lower forced vital capacity, last pCO2, bicarbonate, and base excess, and higher differences in pCO2, were associated with an increase in the length of hospital stay.

**Conclusions:**

Noninvasive positive pressure ventilation application shortly after symptom onset and ALS diagnosis in patients with CO_2_ retention and reduced forced vital capacity can be considered for successful adherence. Adherence to noninvasive positive pressure ventilation may result in reduced tracheostomy conversion rates and prolonged tracheostomy-free survival.

## Introduction

Amyotrophic lateral sclerosis (ALS) is a neurological disorder characterized by the progressive deterioration of motor function resulting from the degeneration of the motor neurons. In patients with ALS, respiratory failure, caused by respiratory muscle weakness with disease progression, represents a major cause of mortality [1]. Thus, evaluation and management of respiratory muscle dysfunction are crucial in clinical settings. Measures of respiratory muscle strength, such as forced vital capacity and peak expiratory flow, are useful in monitoring the aggravation of respiratory muscle weakness; however, no single test of respiratory function can reliably predict the onset of respiratory failure [2, 3].

Noninvasive positive pressure ventilation (NIPPV) has been generally recommended for ALS patients with chronic respiratory failure over the past few decades. Patients receiving NIPPV experience relief of symptoms related to respiratory muscle weakness, including dyspnea, orthopnea, fatigue, morning headache, and sleep disturbance [4, 5]. According to previous studies, NIPPV improves the quality of life, cognitive performance, and survival by up to 18 months; moreover, it curbs the decline of pulmonary function [5–8]. The current guidelines from the American Academy of Neurology recommend the application of NIPPV at the onset of respiratory symptoms or when the forced vital capacity (FVC) falls below 50% of the predicted value [9]. In contrast, the European Federation of Neurological Societies recommends the application of NIPPV when a patient is symptomatic or has FVC < 80%, SNIP < 40 cmH_2_O, significant nocturnal desaturation, or partial pressure of carbon dioxide (pCO_2_) measured in the morning > 45 mmHg [10]. Furthermore, a recent placebo-controlled trial including patients with ALS demonstrated that NIPPV attenuated a decline in FVC when implemented at FVC > 80% [11]. However, there is still no consensus on the ideal timing or indication for NIPPV initiation.

Despite the improvement in survival and quality of life, analysis of the ALS Care Database, a large national observational database of patients with ALS and providers in North America since 1996, showed that only 36% of patients with FVC < 50% used NIPPV [12, 13]. Moreover, noninvasive ventilation was recommended in 23% of patients but was applied in only 9.5% in a recent multi-center cross-sectional study in Korea [14]. Due to the progressive worsening of respiratory function, patients often use NIPPV for several hours a day until total dependence; thus, improving adherence when initiating NIPPV in patients with ALS remains challenging.

Adherence to NIPPV can be influenced by the site of onset, bulbar symptoms, and cognitive impairment [15–17]. However, only a limited number of studies have been published on the factors associated with NIPPV adherence in patients with ALS, and most were conducted in Western countries. Moreover, recent studies demonstrated that the initiation of NIPPV at an earlier stage of the disease prolongs survival [6, 11], but its effect on NIPPV adherence is controversial. Therefore, this study aimed to investigate the factors associated with optimal NIPPV initiation and adherence and assess the long-term effects of NIPPV adherence in patients with ALS who were admitted to a tertiary hospital. The factors that affected the length of hospital stay (LOS) in patients with ALS who initially received NIPPV were also assessed.

## Materials and methods

### Study design and participants

This was a retrospective cohort study of patients with ALS admitted to a tertiary hospital for NIPPV between January 2005 and September 2021. The inclusion criteria were as follows: 1) patients fulfilling the El Escorial World Federation criteria for probable or definite ALS which was confirmed by both neurologists and rehabilitation physicians; 2) patients aged ≥ 19 years; 3) patients who underwent blood gas analysis, including arterial or venous blood gas analyses, before NIPPV application; and 4) patients with FVC < 50% of the predicted value [9] or pCO_2_ measured in the morning > 45 mmHg [10]. The exclusion criteria were as follows: 1) patients with a history of using NIPPV or currently using NIPPV; 2) patients from outpatient settings; 3) patients with a history of asthma or COPD; 4) patients with no record of NIPPV application time; and 5) patients diagnosed with other motor neuron disease. The study protocol was approved by the institutional review board or ethics committee of the Seoul National University Bundang Hospital (B-2210-786-107). The need for informed consent was waived because of the retrospective nature of the study. This study was conducted in accordance with the 1975 Declaration of Helsinki.

### Clinical and pulmonary function parameters

Demographic characteristics, including age at NIPPV application, sex, body mass index (BMI), use of riluzole, and the use of percutaneous endoscopic gastrostomy (PEG), were recorded. According to the initial symptoms and disease history, the site of onset was classified as bulbar or limb onset. Patients who presented with bulbar and limb symptoms were classified as having bulbar onset.

The ALS Functional Rating Scale-Revised (ALSFRS-R), a validated 48-point scale that measures bulbar, motor, and respiratory function, was used at the time of NIPPV initiation [18]. Furthermore, the rate of disease progression (ΔFS) was calculated at baseline using the following formula [19]:

$$\Delta FS=\frac{48-ALSFRS-R\;score\;at\;the\;time\;of\;survey}{duration\;from\;onset\;to\;time\;of\;survey\;(months)}$$

The neurophysiological index (NPI), a biomarker of lower motor neuron injury, was measured using standard neurophysiology equipment [20]. The NPI of the abductor digiti minimi muscle (ADM) was calculated using the following formula and averaged for both hands:

$$NPI=\frac{CMAP\;amplitude\;(ADM)\times F\;wave\;frequency\;(ADM)}{Distal\;motor\;latency\;(ADM)}$$

Ten consecutive stimulation trains were performed with supramaximal stimulation for F-wave frequency. The medical research council (MRC) sum score was summated from six muscles on both sides, ranging from 0 to 60, to assess limb power.

Data regarding variables, such as pH, pCO_2_, HCO_3_-, and base excess for arterial or venous blood gas results, were collected before and after NIPPV application. For patients who underwent blood gas analysis more than once before or after NIPPV application, blood gas analyses just before NIPPV application and before discharge were selected as the initial and final blood gas analysis parameters, respectively. FVC and peak expiratory flow rate (PEF) measured within 6 months prior to NIPPV application were set as the pulmonary function parameters. The incidence of nocturnal desaturation events, defined as oxygen saturation (SpO2) < 90%, was also assessed by a nocturnal oximeter. The length of hospital stay (LOS) was defined as the interval between admission and hospital discharge and was calculated as the number of midnight hours between admission and discharge from the hospital. The time of diagnosis was defined as the time of formal diagnosis based on the El Escorial World Federation criteria; the time between diagnosis and NIPPV application was estimated. Considering the diagnostic delay, the time between symptom onset and NIPPV application was also analyzed.

### Outcomes

The primary outcome of this study was adherence to NIPPV. Adherence to NIPPV was defined as its use for ≥ 2 hours per day. Although previous studies defined adherence to NIPPV as > 4 hours per day [21, 22], a recent study showed that there was a difference in survival between the NIPPV < 1 hour per day group and NIPPV ≥ 1 hour per day group [23]. Therefore, considering the need for NIPPV adaptation with a shorter length of stay under tertiary hospital conditions, adherence to NIPPV was newly defined as a practical criterion. Non-adherence to NIPPV application was defined as its use for < 2 hours per day or refusal to use NIPPV during hospitalization or conversion to invasive positive pressure ventilation (IPPV) via tracheostomy when severe pneumonia or acute respiratory failure occurred. Since most patients used NIPPV intermittently at the time of NIPPV initiation, each application time was recorded according to the caregiver’s report and by the responsible nurse, and the total daily application time of NIPPV was calculated. As in previous studies, patients were additionally divided into two subgroups based on whether they used NIPPV for > 4 hours per day, and subgroup analysis was performed to demonstrate the reliability as a sensitivity analysis.

To analyze the long-term effects in the groups, the conversion rate of NIPPV to IPPV, duration between NIPPV and tracheostomy, duration of survival from NIPPV to death, and duration of tracheostomy-free survival were assessed. Tracheostomy-free survival was defined, as in previous studies, as the combined endpoint of death or tracheostomy [24–26], and it was calculated by the number of months from the initiation of NIPPV to death or tracheostomy. Patients who survived without tracheostomy were censored at the time of the last follow-up. Patients who were lost to follow-up were traced via telephone calls.

### Statistical analysis

Proportions and means with standard deviations were calculated for the demographic variables and baseline patient characteristics. To determine the differences in the factors and long-term effects of NIPPV between the adherent and non-adherent groups and the subgroups, an independent t-test was used for normally distributed data, and the Mann–Whitney U test was used for non-normally distributed data. A chi-square test was performed to compare the gender, site of onset, use of riluzole and PEG, King’s stage, and the rate of conversion to IPPV among the groups. Furthermore, the relationship between adherence to NIPPV and various factors was analyzed using the univariate logistic regression analysis. The effects of individual prognostic factors on the duration between NIPPV and tracheostomy, tracheostomy-free survival, and duration of survival from NIPPV to death were determined using the Kaplan–Meier life table method. Correlations between LOS and continuous variables were analyzed using Pearson’s correlation analysis. Mann–Whitney U test was used to assess the correlation between LOS and ranked variables. All analyses were performed using IBM SPSS Statistics for Windows, version 27.0, and statistical significance was set at p < 0.05.

## Results

### Demographic and clinical characteristics

Fifty-one patients with ALS were included in this study. The main demographic and clinical characteristics of patients are presented in Table 1. The average patient age was 66 years, and 54.9% of the patients were female. The sites of onset were limb (34 patients, 66.7%) and bulbar (17 patients, 33.3%). The mean durations of NIPPV administration from diagnosis and onset were 14 months and 29 months, respectively. None of the patients had a history of chronic lung disease, obstructive sleep apnea, or heart failure. According to 2 hours/day NIPPV-use criteria, twenty-three patients adhered to NIPPV, whereas 28 patients did not adhere to NIPPV. No significant differences were found between the adherent and non-adherent groups with regard to age, sex, or site of onset (p>0.05). In the non-adherent group, eight patients were converted to invasive positive pressure ventilation via tracheostomy due to respiratory failure during hospitalization, and three patients refused to undergo NIPPV and were discharged without its use. Demographic and clinical characteristics based on 4 hours/day NIPPV-use criteria is also shown in Table 2.

**Table 1:** Demographic and clinical characteristics according to 2 hours/day NIPPV-use criteria.

	Total participant (n = 51)	Adherent group (NIPPV≥2h/day) (n = 23)	Non-adherent group (NIPPV<2h/day) (n = 28)	p-value
Age (years)	66.97 ± 10.49	66.50 ± 9.55	67.36 ± 11.19	0.629
BMI (kg/m^2^)	21.41 ± 7.48	23.03 ± 10.43	20.06 ± 3.31	0.161
Sex				
Male	22 (43.1%)	9 (39.1%)	13 (46.4%)	0.438
Female	29 (56.8%)	14 (60.9%)	15 (53.6%)	
Site of onset				
Bulbar	17 (33.3%)	8 (34.7%)	9 (32.1%)	0.802
Limb	34 (66.7%)	15 (65.3%)	19 (67.9%)	
King’s stage				
Stage 1	3 (5.8%)	3 (13.0%)	0 (0%)	0.002
Stage 2	20 (39.2%)	13 (56.5%)	7 (25%)	
Stage 3	10 (19.6%)	5 (21.7%)	5 (17.9%)	
Stage 4	18 (35.2%)	2 (8.6%)	16 (57.1%)	
Time from diagnosis to NIPPV application (months)	14.12 ± 26.44	5.39 ± 10.60	21.29 ± 32.93	0.031
Time from onset to NIPPV application (months)	28.51 ± 27.43	19.91 ± 15.04	35.57 ± 33.08	0.041
FVC %	56.68 ± 19.76	56.18 ± 22.77	57.29 ± 16.18	0.879
PEF %	61.80 ± 20.02	67.25 ± 21.10	55.08 ± 17.97	0.105
Initial blood gas analysis parameter				
pCO_2_ (mmHg)	55.33 ± 10.78	54.58 ± 7.94	55.93 ± 12.76	0.659
HCO_3_- (mmol/l)	31.16 ± 5.99	31.60 ± 3.54	30.80 ± 7.57	0.646
Base excess (mmol/l)	5.25 ± 3.07	5.04 ± 2.82	5.42 ± 3.36	0.672
pH ^a^	7.39 (7.36–7.40)	7.39 (7.37–7.40)	7.39 (7.36–7.41)	0.857
Difference of pCO_2_ (mmHg) ^a^	3.3 (0.5–10.5)	2.6 (0.5–9.0)	3.9 (0.5–12.7)	0.697
LOS (days) ^a^	6.0 (4.0–12.5)	7.0 (5.0–9.0)	6.0 (3.0–18.0)	0.530
ALSFRS-R	20.31 ± 11.85	24.21 ± 12.48	17.22 ± 10.96	0.108
Bulbar	6.69 ± 4.07	9.00 ± 3.46	4.89 ± 3.77	**0.004**
Motor	7.53 ± 7.19	9.36 ± 7.43	6.11 ± 7.08	0.221
Respiratory	6.06 ± 3.40	5.86 ± 3.51	6.22 ± 3.51	0.771
ΔFS	0.84 ± 0.50	0.83 ± 0.54	0.86 ± 0.51	0.924
NPI	3.74 ± 4.41	5.97 ± 5.60	1.92 ± 1.96	0.003
MRC sum score	35.96 ± 16.33	39.83 ± 15.94	34.19 ± 16.43	0.264
Desaturation event	16 (31.4%)	7 (30.4%)	9 (32.1%)	0.896
Riluzole	28 (54.9%)	10 (43.5%)	18 (64.3%)	0.244
PEG	16 (31.4%)	5 (21.7%)	11 (39.3%)	0.179

a: Data are expressed as the median (interquartile range).

NIPPV, noninvasive positive-pressure ventilation; FVC, forced vital capacity; PEF, peak expiratory flow rate; LOS, length of stay; ALSFRS-R, Amyotrophic Lateral Sclerosis Functional Rating Scale-Revised; ΔFS, ALSPRS-R progression rate calculated from disease-onset to baseline; NPI, neurophysiological index; MRC, Medical Research Council; BMI, body mass index; PEG, percutaneous endoscopic gastrostomy

**Table 2:** Demographic and clinical characteristics according to 4 hours/day NIPPV-use criteria.

	Adherent group (NIPPV≥4h/day) (n = 15)	Non-adherent group (NIPPV<4h/day) (n = 36)	p-value
Age (years)	65.26 ± 10.24	67.68 ± 10.51	0.495
BMI (kg/m^2^) ^a^	22.7 (19.3–23.7)	20.5 (18.2–23.0)	0.151
Sex			
Male	6 (40%)	17 (47.2%)	0.637
Female	9 (60%)	19 (52.8%)	
Site of onset			
Bulbar	7 (46.7%)	12 (33.3%)	0.370
Limb	8 (53.3%)	24 (66.7%)	
King’s stage			
Stage 1	2 (13.3%)	1 (2.7%)	0.044
Stage 2	7 (46.6%)	13 (36.1%)	
Stage 3	4 (26.6%)	6 (16.6%)	
Stage 4	2 (13.3%)	16 (44.4%)	
Time from onset to NIPPV application (months)	18.07 ± 15.26	32.86 ± 30.26	0.025
FVC % ^a^^,^ ^b^	49.5 (46.5–73.5)	53.5 (44.25–71.25)	0.858
PEF % ^c^	66.50 ± 25.67	56.35 ± 21.39	0.261
Initial blood gas analysis parameter			
pCO_2_ (mmHg) ^a^	54.7 (48.2–60.4)	52.0 (46.4–63.5)	1.000
HCO_3_- (mmol/l)	31.61 ± 3.87	30.97 ± 6.79	0.734
Base excess (mmol/l)	5.05 ± 3.11	5.33 ± 3.14	0.774
pH ^a^	7.38 ± 0.03	7.38 ± 0.04	0.823
Difference of pCO_2_ (mmHg) ^a^	5.92 (0.75–9.50)	3.25 (0–10.73)	0.809
LOS (days) ^a^	7.0 (5.0–9.5)	6.0 (4.0–14.0)	0.603
ALSFRS-R	20.67 ± 11.80	20.13 ± 12.32	0.912
Bulbar	8.44 ± 3.71	6.00 ± 4.17	0.135
Motor	7.22 ± 6.70	7.65 ± 7.66	0.884
Respiratory	5.00 ± 3.74	6.48 ± 3.33	0.284
ΔFS	0.98 ± 0.61	0.79 ± 0.47	0.364
NPI	4.57 ± 3.88	3.37 ± 4.70	0.391
MRC sum score	35.58 ± 16.54	36.84 ± 16.44	0.822
Desaturation event	5 (33.3%)	11 (30.6%)	0.846
Riluzole	5 (33.3%)	23 (63.8%)	0.236
PEG	4 (26.7%)	12 (33.3%)	0.640

a: Data are expressed as the median (interquartile range).

b: n = 11 for NIPPV≥4h/day group, n = 20 for NIPPV<4h/day group

c: n = 10 for NIPPV≥4h/day group, n = 19 for NIPPV<4h/day group

NIPPV, noninvasive positive-pressure ventilation; FVC, forced vital capacity; PEF, peak expiratory flow rate; LOS, length of stay; ALSFRS-R, Amyotrophic Lateral Sclerosis Functional Rating Scale-Revised; ΔFS, ALSPRS-R progression rate calculated from disease-onset to baseline; NPI, neurophysiological index; MRC, Medical Research Council; BMI, body mass index; PEG, percutaneous endoscopic gastrostomy

### Factors associated with adherence to NIPPV

Based on 2 hours/day NIPPV-use criteria, the duration between the diagnosis of ALS and NIPPV application (adherent group: 5.39 ± 10.60 months vs. non-adherent group: 21.29 ± 32.93, p = 0.031) and between symptom onset and NIPPV application (adherent group: 19.91 ± 15.04 months vs. non-adherent group: 35.57 ± 33.08, p = 0.041) was significantly shorter in the adherent than that in the non-adherent group. Patients in the non-adherent group demonstrated a significantly lower NPI value (adherent group: 5.97 ± 5.60 vs. non-adherent group: 1.92 ± 1.96, p = 0.003) and ALSFRS-R bulbar subscore (adherent group: 9.00 ± 3.46 vs. non-adherent group: 4.89 ± 3.77, p = 0.004) than that of those in the adherent group. Moreover, the significant difference in King’s stage distribution between the groups was demonstrated, which revealed the higher proportion of advanced stages in the non-adherent group (p = 0.002). However, FVC was not significantly lower in the adherent group than that in the non-adherent group (p = 0.879) (Table 1).

The comparison based on 4 hours/day NIPPV-use criteria showed a similar tendency. No significant differences were found between the subgroups with regard to age, sex, site of onset, or FVC (Table 2). The duration between diagnosis of ALS and NIPPV application and between symptom onset and NIPPV application was significantly shorter in the NIPPV ≥ 4 hours per day subgroup than that in the NIPPV < 4 hours per day subgroup. Also, a statistically significant difference in King’s stage distribution was observed between the subgroups, indicating a greater prevalence of advanced stages in the NIPPV < 4 hours per day subgroup (p = 0.044).

Univariate analysis based on 2 hours of NIPPV application revealed that the factors influencing adherence to NIPPV were the duration between diagnosis and NIPPV application (OR 0.93, 95% CI: 0.87–0.99, p = 0.031), duration between onset and NIPPV application (OR 0.96, 95% CI: 0.92–1.00, p = 0.045), ALSFRS-bulbar subscore (OR 1.35, 95% CI: 1.08–1.69, p = 0.010), and NPI (OR 1.62, 95% CI: 1.19–2.20, p = 0.002) (Table 3). Univariate analysis based on 4 hours of NIPPV application demonstrated that the factors influencing adherence to NIPPV were the duration between diagnosis and NIPPV application (OR 0.799, 95% CI: 0.667–0.957, p = 0.015), duration between onset and NIPPV application (OR 0.952, 95% CI: 0.906–1.001, p = 0.048).

**Table 3:** Predictive model for adherence to NIPPV according to 2 and 4 hours/day NIPPV-use criteria.

	Univariate analysis (2 hours NIPPV application)		
Variables	OR	95% CI	p-value
Age	0.992	0.941–1.046	0.772
Limb onset	0.864	0.276–2.701	0.802
Male	1.556	0.509–4.758	0.439
Initial pCO2	0.988	0.938–1.041	0.652
FVC	0.997	0.961–1.034	0.874
Time from diagnosis to NIPPV application (months)	0.930	0.871–0.993	0.031
Time from onset to NIPPV application (months)	0.959	0.920–0.999	0.045
ALSFRS-R			
Total score	1.055	0.989–1.125	0.105
Bulbar subscore	1.348	1.075–1.690	0.010
Δ FS	0.932	0.233–3.736	0.921
NPI	1.617	1.191–2.195	0.002
	Univariate analysis (4 hours NIPPV application)		
Variables	OR	95% CI	p-value
Age	0.978	0.924–1.036	0.453
Limb onset	1.750	0.521–5.978	0.372
Male	0.745	0.219–2.531	0.637
Initial pCO2	0.989	0.934–1.048	0.719
FVC	0.988	0.951–1.028	0.558
Time from diagnosis to NIPPV application (months)	0.799	0.667–0.957	0.015
Time from onset to NIPPV application (months)	0.952	0.906–1.001	0.048
ALSFRS-R			
Total score	1.004	0.940–1.072	0.908
Bulbar subscore	1.175	0.949–1.456	0.140
Δ FS	2.005	0.456–8.820	0.358
NPI	1.059	0.927–1.210	0.399

NIPPV, noninvasive positive-pressure ventilation; FVC, forced vital capacity; NIPPV, noninvasive positive-pressure ventilation; FVC, forced vital capacity; ALSFRS-R, Amyotrophic Lateral Sclerosis Functional Rating Scale-Revised; ΔFS, ALSPRS-R progression rate calculated from disease-onset to baseline; NPI, neurophysiological index

### Long-term effects of NIPPV adherence

**Fig 1:**
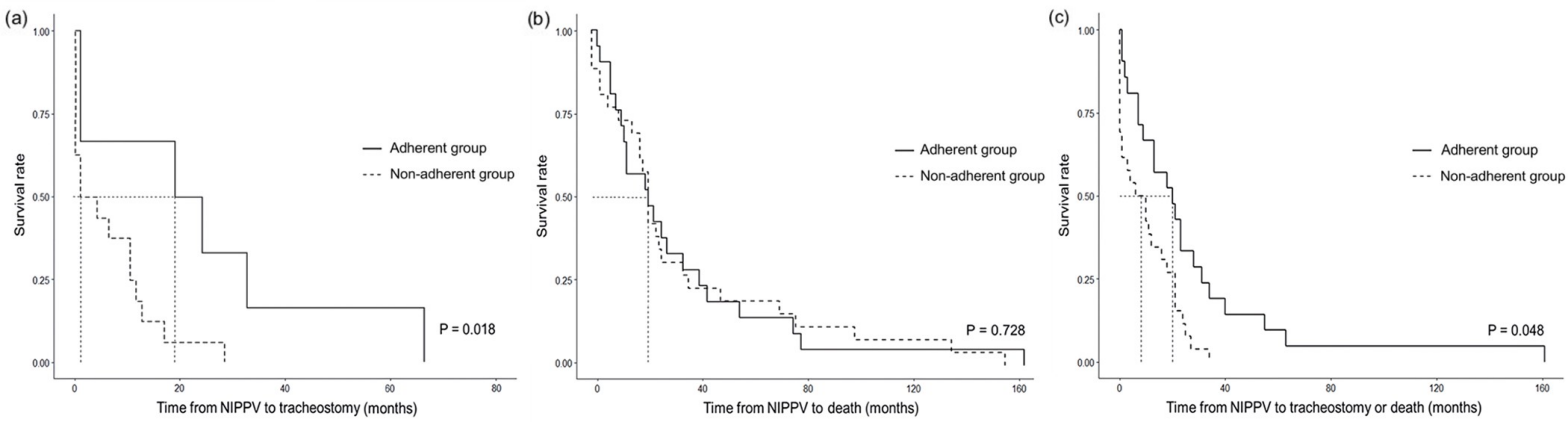
Survival rates according to the duration from noninvasive positive pressure ventilation (NIPPV) to tracheostomy or death in patients with amyotrophic lateral sclerosis (ALS) in the adherent and non-adherent groups. Kaplan–Meier survival curves of patients with ALS demonstrated a significant difference in the duration between NIPPV and tracheostomy (p = 0.018, log-rank test) (a) and tracheostomy-free survival between the adherent and non-adherent groups (p = 0.048, log-rank test) (c), although there is no difference in survival between the groups (p = 0.728, log-rank test) (b).

Four patients were lost to follow-up, and the mean follow-up period from NIPPV application to death or last contact was 35.2 months in 47 patients. The most frequent cause of death was respiratory failure as a consequence of weakness of respiratory muscles (94.8%), while a single fatality was attributed to pneumonia (2.6%) and another to cardiovascular causes (2.6%). Twenty-two patients (46.8%) converted to IPPV via tracheostomy after NIPPV application. Compared to the non-adherent group, the adherent group was less likely to progress to IPPV (28.6% vs. 61.5%, p = 0.024) based on the 2 hours per day NIPPV-use criteria. Furthermore, the tracheostomy-free duration of survival was significantly longer in the adherent group (median: 20 months [interquartile range, 7.0–31.0]) than that in the non-adherent group (median: 8 months [interquartile range, 0–20.2]) (p = 0.010) (Table 4). Based on the 4 hours per day NIPPV-use criteria, the adherent group was less likely to progress to IPPV compared to the non-adherent group (23.1% vs. 55.9%, p = 0.044). The Kaplan–Meier survival curves also showed a significant difference in the duration from NIPPV to tracheostomy (p = 0.018) and tracheostomy-free survival between the adherent and non-adherent groups (p = 0.048), although no difference in survival from NIPPV to death was noted between adherent and non-adherent groups (p = 0.728) (Fig 1).

**Table 4:** Differences in conversion to IPPV via tracheostomy between the adherent and non-adherent groups based on the 2 and 4 hours/day NIPPV-use criteria.

	Adherent group (NIPPV≥2h/day)	Non-adherent group (NIPPV<2h/day)	p-value
Tracheostomy-free survival duration (months)	20.0 (7.0–31.0)	8.0 (0–20.2)	0.010
Conversion to IPPV			
Yes	6 (28.6%)	16 (61.5%)	0.024
No	15 (71.4%)	10 (38.5%)	
	Adherent group NIPPV≥4h/day	Non-adherent group NIPPV<4h/day	p-value
Tracheostomy-free survival duration (months)	18.0 (7.0–28.0)	10.5 (1.0–21.8)	0.197
Conversion to IPPV			
Yes	3 (23.1%)	19 (55.9%)	0.044
No	10 (76.9%)	15 (44.1%)	

IPPV, invasive positive pressure ventilation; NIPPV, noninvasive positive-pressure ventilation

**Fig 2:**
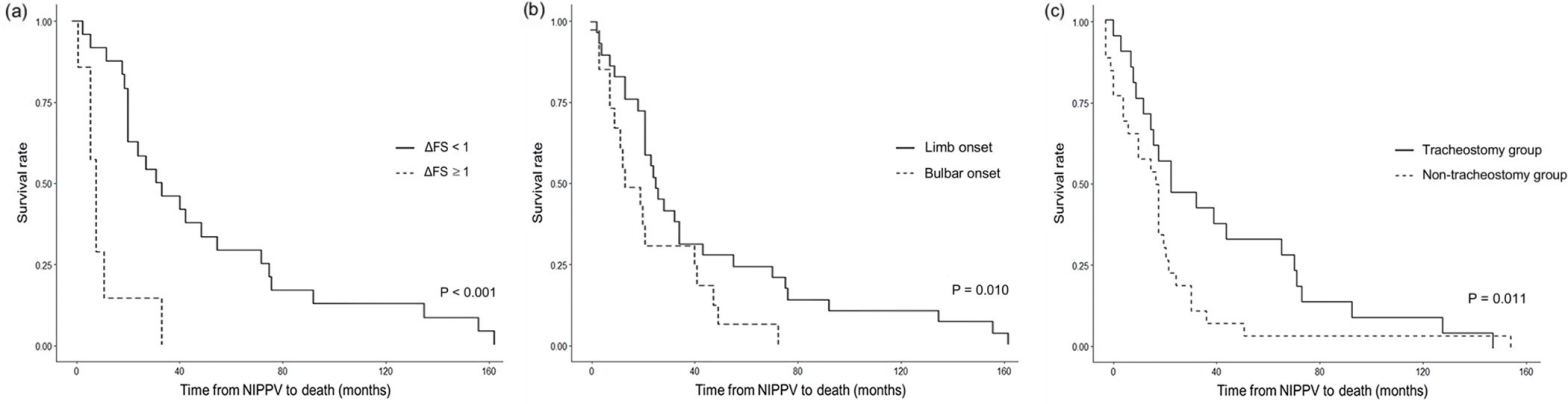
Survival rates by time from noninvasive positive pressure ventilation (NIPPV) to death in patients with amyotrophic lateral sclerosis (ALS) according to the site of onset, progression rate, and the presence of tracheostomy. Kaplan–Meier survival curves of patients with amyotrophic lateral sclerosis show a significant difference in the survival between the progression rate (ΔFS) ≥ 1 group and ΔFS < 1 group (p<0.001, log-rank test) (a), bulbar onset and limb onset (p = 0.010, log-rank test) (b), and tracheostomy and non-tracheostomy groups (p = 0.011, log-rank test) (c).

In terms of prognostic predictors of survival, IPPV through tracheostomy significantly prolonged survival compared to the non-tracheostomy group. Additionally, the group with limb onset ALS and slow progression rate (ΔFS < 1) showed significantly prolonged survival than that of the group with bulbar onset ALS and fast progression rate (ΔFS ≥ 1) (Fig 2).

### Factors correlated with length of hospital stay for NIPPV initiation

**Fig 3:**
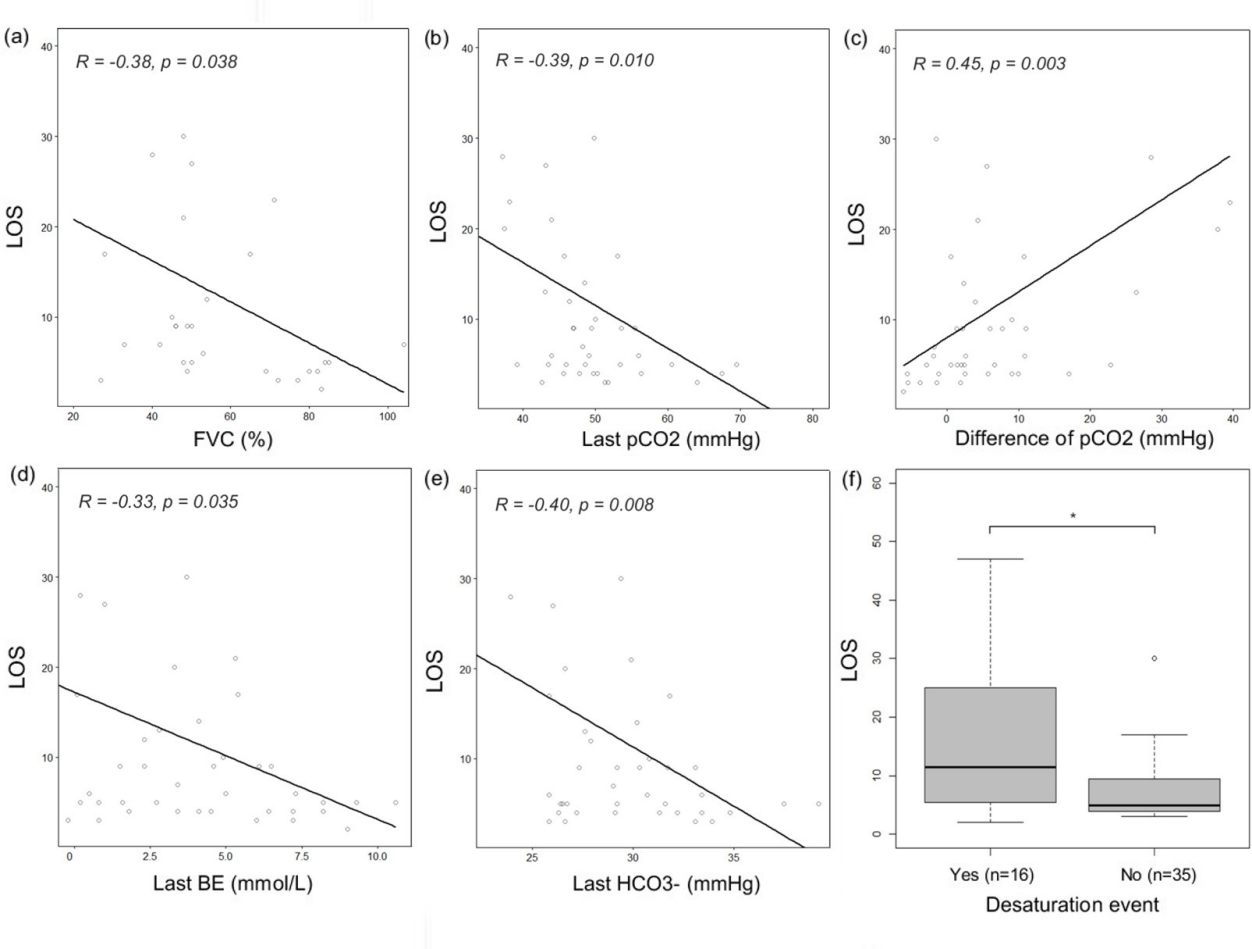
The length of hospital stay (LOS) according to forced vital capacity (FVC), blood gas analysis parameters, and desaturation event. (a) The scatter plot of Pearson correlation analysis shows a negative correlation between LOS and FVC (r = -0.38, p = 0.038). (b, d, e) LOS shows negative correlations with the levels of partial pressure of carbon dioxide (pCO_2_) (r = -0.39, p = 0.010), base excess (r = -0.33, p = 0.035), and HCO_3_^-^(r = -0.40, p = 0.008) which was measured before discharge. (c) LOS shows positive correlations with the difference in the initial and final pCO_2_ (r = 0.45, p = 0.003). (f) The median LOS is significantly longer in the patients who experienced a desaturation event (oxygen saturation [SpO_2_] <90%) (11.5 days) than that in patients who did not (5 days, p = 0.019).

Sixteen patients experienced a desaturation event during hospitalization. LOS in patients who experienced a desaturation event (median: 11.5 days [interquartile range, 5.75 to 24.0]) was longer than that in those who did not experience a desaturation event (median: 5 days [interquartile range, 4.0–9.5]) (p = 0.019). LOS was also significantly increased in patients with lower FVC (r = -0.38, p = 0.038). Blood gas analysis parameters, including the last measured value, such as the differences in pCO_2_ (r = 0.45, p = 0.003), last pCO_2_ (r = -0.39, p = 0.010), last base excess (r = -0.33, p = 0.035), and last HCO_3_- (r = -0.40, p = 0.008), also showed a significant correlation with LOS (Fig 3). However, the blood gas analysis parameters that were measured initially did not have a significant correlation with LOS (p>0.05).

## Discussion

This retrospective cohort study revealed that the shorter duration from symptom onset and diagnosis of ALS to NIPPV application was associated with increased adherence to NIPPV. The duration between diagnosis and NIPPV application and between symptom onset and NIPPV in the adherent group (NIPPV ≥ 2 h/day) was shorter than that in the non-adherent group (NIPPV < 2 h/day). The same result was obtained on applying NIPPV for 4 hours per day as the criteria. Thus, the initiation of NIPPV shortly after ALS symptom onset and diagnosis may result in successful NIPPV adherence. Since adherence to NIPPV has been suggested as a prognostic factor for survival in ALS [27], it could help determine the timing of NIPPV initiation in outpatient counseling.

Lower NPI values at baseline were associated with poor adherence to NIPPV in our study, indicating a greater degree of lower motor neuron dysfunction in the non-adherent group with reduced NPI values [28, 29]. However, NPI was applied only at the ulnar nerve, and it relies on the absence of ulnar entrapment neuropathies; therefore, a more accurate tool to detect and quantify the severity of lower motor neuron dysfunction is required to explain some of the findings of the present study.

Although the current guidelines recommend NIPPV initiation based on FVC [9, 10], there was no significant difference between the adherent and non-adherent groups according to FVC. These findings are consistent with those of a previous study that reported no difference in the adherence and acceptance rates between the low FVC (FVC < 80%) and high FVC groups (FVC > 80%) [22]. It is postulated that the high FVC group refused to apply NIPPV since their respiratory function was not yet severely compromised. However, in this hospital, NIPPV was initiated in most patients primarily based on their respiratory symptoms. As the pulmonary function of half of the patients was not measured just before NIPPV initiation, the causal relationship between pulmonary function and adherence was unclear.

Previous studies have applied the concept of early NIPPV based on the degree of respiratory impairment measured by FVC [26, 30]. Specifically, they considered applying early NIV when FVC was above the specified threshold, rather than below 50% it as stated in the guidelines. However, the current study differs in that it associates the duration from diagnosis or onset to the NIPPV initiation. Nevertheless, this approach suggests that the application of NIPPV should be considered after ALS diagnosis, which is thought to be of significant importance in the clinical setting.

The role of blood gas analysis as a predictor of pulmonary function and survival after NIPPV initiation and as a marker of ventilator compliance has been highlighted [31]. Additionally, hypercapnia, though a relatively late finding, remains a potential indicator for the initiation of NIPPV [32]. However, blood gas analysis parameters measured before NIPPV initiation were not correlated with the success of NIPPV in this study. This may be because most patients (n = 47) showed chronic hypercapnia (mean initial pCO_2_ of total participants; 55.33±10.67) which was high compared to the major guidelines [10, 33], and were symptomatic before NIPPV initiation in the study. Therefore, further studies should include a broader range of variables to determine the association between blood gas analysis and NIPPV adherence.

The majority of patients in the study showed bulbar symptoms (41 patients, 80.3%) or respiratory symptoms (47 patients, 92.2%) at the time of NIPPV application. Accordingly, it appears that the longer the time until NIPPV application is delayed, the worse bulbar or respiratory symptoms are likely to become. Previous studies have also revealed that NIPPV adherence decreased as bulbar symptoms worsened [15, 17]. These bulbar symptoms originate from the disability of the oropharyngeal muscles, which makes swallowing and communication difficult. In particular, upper airway obstructive events, accumulated airway secretions, failure of lip closure and drooling are factors that can impair NIPPV tolerance [34]. Considering this, application of NIPPV shortly after the time of diagnosis or symptom onset may have a positive effect on adherence.

Regarding the 2 hours/day NIPPV-use criteria, it is difficult for patients to have prolonged hospitalization due to the characteristics of a tertiary hospital. However, based on our empirical experience, when NIPPV was applied for more than 2 hours per day for the first time during hospitalization, most patients were able to tolerate and subsequently receive NIPPV for more than 4 hours per day during outpatient follow-up. Moreover, previous studies that used a 4-hour criterion for NIPPV demonstrated the restorative effect of respiratory muscle rest and improved long-term outcomes such as survival and FVC decline rates. Through this study, we provide evidence supporting the efficacy of applying NIPPV for more than 2 hours per day; thus, we believe that this study could suggest as a new standard by demonstrating the improvement in long-term outcomes using the criterion of applying NIPPV for more than 2 hours per day. A previous study demonstrated that the early use of NIPPV for > 4 hours per day significantly prolonged tracheostomy-free survival [26]. The importance of riluzole use, which prolonged tracheostomy-free survival, has also been demonstrated [25, 35]. In this study, the adherent group (NIPPV ≥ 2 h/day) showed a decreased need for tracheostomy (Table 4) and significantly prolonged tracheostomy-free survival (Fig 1), although there was no difference in riluzole use. The use of NIPPV for > 4 hours per day also decreased the conversion rate of NIPPV to IPPV. This finding suggests that adherence to NIPPV has long-term effects on tracheostomy-free survival.

Contrary to expectations, there was no difference in survival between the adherent and non-adherent groups, which may be attributed to the different ratios of conversion to IPPV. In our study, the conversion rate to IPPV was higher (61.5%) in the non-adherent group than that in the adherent group (28.6%). Considering the prolonged survival after IPPV through tracheostomy, the effects of NIPPV adherence on survival were possibly masked by the conversion to IPPV. Furthermore, previous studies demonstrated that the number of patients with ALS who underwent tracheostomy varied, ranging from 11.2 to 31.3% [36–38]. A higher rate of conversion to IPPV through tracheostomy than those in previous studies likely had a greater impact on survival, although tracheostomy worsens the patients’ quality of life.

IPPV through tracheostomy significantly prolonged survival in the tracheostomy group compared to that in the non-tracheostomy group. The median survival after IPPV through tracheostomy was 15 months, which was previously reported to be 8–25 months [37–39]. However, despite the benefits of tracheostomy in terms of survival, the timing and indications for IPPV remain unclear, and various factors, such as caregiver support and cognitive function, should be considered before the initiation of IPPV. ΔFS score ≥ 1 and bulbar-onset disease were also established as potential adverse prognostic biomarkers of survival in the current ALS cohort. These findings are consistent with those of previous studies, which identified ΔFS and the site of onset as prognostic biomarkers [40, 41]. Therefore, the site of disease onset and ΔFS score could be reliable prognostic biomarkers for ALS and could be utilized in patient care planning and stratification tool. LOS in this study (mean LOS; 9.91 ± 9.35) was slightly shorter than that in a previous study, which reported that the mean inpatient stay was approximately 12 days for NIPPV initiation [21]. The factors associated with LOS were desaturation events, FVC, and blood gas analysis parameters (Fig 3). The occurrence of desaturation events during hospitalization was positively correlated with LOS. This finding was consistent with that of previous studies, which suggested that patients with ALS have lengthy and costly hospital admissions due to the high incidence of respiratory failure [42, 43]. Moreover, FVC and LOS had a negative correlation. This was likely because patients with low FVC tended to experience desaturation events during hospitalization (FVC in desaturation event during hospitalization: 50.50 ± 16.27 vs. FVC in non-desaturation event: 60.58 ± 21.15, p = 0.066). Lastly, the blood gas analysis parameters measured immediately before discharge were correlated with LOS, suggesting that the duration of NIPPV application influences respiratory and metabolic physiology.

This study had several limitations. First, respiratory muscle strength based on the maximum inspiratory pressure or sniff nasal inspiratory pressure was not evaluated. Moreover, the reliability of FVC values might be compromised due to patients who were unable to perform the test because of bulbar symptoms, and lack of accurate information about the supine or sitting position during measurement. Second, although home care services were implemented, there was no detailed information regarding NIPPV use after discharge. Thus, long-term adherence to NIPPV could not be evaluated. Third, the information about cognitive function and socioeconomic status such as income and educational status, which were known to be the determinants of NIPPV adherence, was limited in this study. Most of the patients in this study were married and lived with their families (94.1%), who provided caregiving support (88.2%), making it difficult to analyze these factors as determinants of adherence. Finally, the sample size of this study was relatively small, and several outcome parameters could not be assessed in some patients. A larger sample size is required to generalize the results and progress the multivariate regression analysis for predicting the adherence of NIPPV. A multicenter prospective cohort study with long-term follow-up will pave the way for identifying the predictive factors for adherence to NIPPV in patients with ALS.

## Conclusions

NIPPV application shortly after symptom onset and ALS diagnosis in patients with CO_2_ retention and respiratory symptoms may aid in successful NIPPV adherence, taking into account the severity of lower motor neuron dysfunction. Adherence to NIPPV has long-term effects, such as reduced tracheostomy conversion rate and prolonged tracheostomy-free survival. In addition, LOS was positively correlated with desaturation events and negatively correlated with FVC.
